# The efficiency of provincial government health care expenditure after China’s new health care reform

**DOI:** 10.1371/journal.pone.0258274

**Published:** 2021-10-13

**Authors:** Xuesong Guo, Jun Zhang, Zhiwei Xu, Xin Cong, Zhenli Zhu

**Affiliations:** 1 School of Public Policy and Administration of Xi’an Jiaotong University, Xi’an, Shanxi, China; 2 Yantai Affiliated Hospital of Binzhou Medical University, Yantai, Shandong, China; 3 Department of the Quality and Management of the Medical, Yantai Affiliated Hospital of Binzhou Medical University, Yantai, Shandong, China; 4 Department of Communist Youth League of the Medical, Yantai Affiliated Hospital of Binzhou Medical University, Yantai, Shandong, China; 5 Department of Education, Yantai Affiliated Hospital of Binzhou Medical University, Yantai, Shandong, China; Universidad Nacional Autonoma de Nicaragua Leon, NICARAGUA

## Abstract

**Objective:**

We aim to estimate the total factor productivity and analyze factors related to the Chinese government’s health care expenditure in each of its provinces after its implementation of new health care reform in the period after 2009.

**Materials and methods:**

We use the Malmquist DEA model to measure efficiency and apply the Tobit regression to explore factors that influence the efficiency of government health care expenditure. Data are taken from the China statistics yearbook (2004–2020).

**Results:**

We find that the average TFP of China’s 31 provincial health care expenditure was lower than 1 in the period 2009–2019. We note that the average TFP was much higher after new health care reform was implemented, and note this in the eastern, central and western regions. But per capita GDP, population density and new health care reform implementation are found to have a statistically significant impact on the technical efficiency of the provincial government’s health care expenditure (P<0.05); meanwhile, region, education, urbanization and per capita provincial government health care expenditure are not found to have a statistically significant impact.

**Conclusion:**

Although the implementation of the new medical reform has improved the efficiency of the government’s health expenditure, it is remains low in 31 provinces in China. In addition, the government should consider per capita GDP, population density and other factors when coordinating the allocation of health care input.

**Significance:**

This study systematically analyzes the efficiency and influencing factors of the Chinese government’s health expenditure after it introduced new health care reforms. The results show that China’s new medical reform will help to improve the government’s health expenditure. The Chinese government can continue to adhere to the new medical reform policy, and should pay attention to demographic and economic factors when implementing the policy.

## 1. Introduction

It is well known that health care institutions experienced many problems in China after the SARS outbreak in 2003, and that they affected the implementation of health care policy reform. Although China’s provincial health care expenditure increased every year, and the government established a basic medical system that covers both urban and rural residents, high medical costs continue to be a widespread problem. The "China Youth Daily" proposed that unsuccessful Chinese medical reform should be brought to an end. This proposal was published by the Department of Medicine and Health System Reform in 2005, which is accountable to the Development Research Center of the State Council. Health care reform also almost failed in the period 2004–09 because the popular burden of health care became heavier. China therefore launched a new round of health care reform in 2009, which mainly sought to enhance the government’s leadership and increase health care investment. This medical reform program proposed that governments should increase their spending by 850 billion yuan to support medical and health care reform in 2009–2011. As a consequence, the proportion of total health expenditure to gross domestic product (GDP) increased from 4.55 in 2008 to 6.64 in 2019. Government spending on health care grew much faster than GDP, and this underlined the Government’s determination to solve the problem. But the challenges of reform were greater than other fields, as the health care system is complex and reformers must take demographic, economic, political and social factors into account [1]. New health care reform (NHCR) has been implemented for more than ten years. China’s aging population is growing and demand for medical and health care has greatly increased. The new rural medical insurance that the Chinese government introduced in 2009 covers more people, and has further stimulated popular demand for medical treatment [2]. Although the Chinese government has increased its investment in health care, the country’s medical and health care service supply has been unable to meet rapidly growing demand, which has produced supply-demand imbalance, tensions between doctors and patients and rapidly rising medical costs [3]. The neglect of the efficiency of government health care expenditure has created many difficulties for the optimization of health care expenditure. The main problems include: (1) insufficient and unbalanced health care investment in the eastern, central and western regions; (2) the distribution of health care resources, which have taken the form of "inverted pyramid" that mainly concentrates high-quality health care resources in developed regions; (3) a ‘mismatch’ between substantial increases in health care expenditure and patient access to medical treatment. As a result, the doctor-patient relationship has become more difficult.

In addition, the COVID-19 pandemic presents a huge challenge to the country’s health care provision and governance. It is essential for the country’s health care services to improve the efficiency of government health expenditure, as this will help to solve existing health care problems. While it is therefore time to evaluate the government health care expenditure of the new health care reform, domestic and foreign scholars have not systematically evaluated the efficiency of this round of new health reform.

Health care expenditure has therefore been recognized as a growing burden on the level of economic development in most nations and as a principal factor in the determination of country development balance [4]. The performance evaluation of many sectors, including health care systems, were challenging and provided a useful decision-making tool that sought to optimize resource use [5]. Research into the health care system’s output efficiency mainly falls into two categories: the first is the traditional parameter-type method based on the production function, and the second is non-parametric data envelopment analysis (DEA). But most studies try to evaluate and measure the health care system’s relative efficiency by using cross-sectional data [6–19]; in contrast, few studies have aimed to analyze the efficiency of government health care expenditure, and this is especially true of the analysis of panel data.

A number of previous studies focus on the efficiency of the health care system or hospitals. Qian Li assesses the efficiency of county public hospitals in Shandong Province after China’s NHCR and compares the efficiency of hospitals with different bed sizes [6]. Xu Meimei evaluates the scientific research efficiency of tertiary hospitals in China and focuses on the decision-making of scientific research departments in China [20]. Jing Liu uses a three-level growth model to identify determinants that contribute to efficiency changes in public county-hospitals over time [21]. Gong Guangwen uses network DEA to evaluate the two substage efficiencies of China’s healthcare system after the implementation of health care reform [1]. RY Gai assesses trends in the productive efficiency of China’s county hospitals during the economic transition by using data from the period 1993–2005 [22]. Haniye Sadat Sajadi measures efficiency and productivity changes in the Iranian health care system and also assesses its progress towards UHC by making a comparison with 36 upper-middle-income countries [23]. Garcia-Cornejo uses public data taken from 159 hospitals in the Spanish National Health System (NHS) in the period 2010–13, and analyzes if the development of hospital cost systems (CS) implemented by the Spanish Regional Health Services (RHS) has affected hospital efficiency [24].

Mehmet Top measures the health care system efficiency of 36 African countries and compares efficiency levels between countries [25]. Berger analyzes the association between Soft budget constraints (SBCs) and hospital efficiency change in a setting where there is with negligible risk of hospital closure. He applies a two-stage analysis based on bias-corrected non-radial input-oriented DEA and ordinary least squares regression (OLSR) [26]. Babalola assesses the technical efficiency (TC) and productivity of public district hospitals in South Africa’s KwaZulu-Natal province [27]. Vidhya analyzes changes in the TFP index of an Indian state’s public district hospitals in the period 2013–2017 with the aim of identifying their efficiency patterns after the implementation of HMIS (Health Management Information System) [28].

In China, medical and health care institutions are still dominated by the government, which has taken a significant step towards accelerating the decision-making process with the intention of improving the performance of health care systems in different provinces. Taking into account the unbalanced allocation of health resources and the increasingly obvious contradiction between supply and demand, it is extremely important to effectively improve the total factor productivity (TFP) of government health care expenditure and make full use of regional health resource, as this will help to meet growing resident demand for health services. In this article, factors that influence government health care expenditure are therefore analyzed with the intention of providing evidence-based policy insights that will contribute to improvements in health care efficiency. Since 1994, China has implemented financial decentralization. Under the context of financial decentralization, China’s central government health expenditure is mainly borne by the local government. This study uses the non-parametric data envelope analysis (DEA) method to measure the efficiency of China’s provincial government health care expenditure in the first stage. It then uses the restricted variable Tobit random effect panel model to do regression analysis, and this make it possible to find factors that may affect local government health care expenditure efficiency. This study uses two-stage analysis, to try to answer the following questions: First, how does the efficiency of China’s local government health care expenditure? Is there a difference in efficiency in different regions? What factors affect the efficiency of government health expenditures?

## 2. Materials and methods

### 2.1 The Malmquist DEA model

DEA has been widely used to evaluate productivity and efficiency in various fields [29–35], especially seeks to efficiently examine the efficiency of health care providers [36–38]. This study combines the BCC (Banker Charnes Cooper) model [39] with the Malmquist Index Model and undertakes data envelopment analysis (DEA) [40] with the aim of evaluating and analyzing the efficiency of provincial government health care expenditure in China. DEA is a non-parametric technical efficiency evaluation method: it does not only calculate the relative efficiency score of decision-making units (DMU), but also highlights aspects of excess input or insufficient output for DMUs that are not effective in DEA, and it enables decision-makers to rely on the data when adjusting structure and improving operations [41,42]. This method uses linear programming to construct an efficient convex production frontier, and identifies the relative level of efficiency by comparing it against this frontier. DEA can be divided into three categories: a single-stage CCR-DEA model based on the assumption of constant return on scale [41]; a single-stage BCC-DEA model based on variable return on scale [42]; and a Malmquist DEA model based on intertemporal analysis [40].

The DEA-Tobit two-stage framework can be used to analyze the influence of various economic, social and policy factors on the efficiency of government expenditure, and it can be implemented on the basis of calculating the efficiency of government expenditure. In addition, the analytical framework has several technical advantages: first, the output of government health care expenditure includes multiple aspects, and the DEA method is uniquely able to deal with the efficiency accounting problem of multi-input and output; second, DEA is a nonparametric estimation method, which means it can effectively avoid the problem of model setting error; finally, the Tobit Regression Model can overcome the interception problem of efficiency distribution. Whereas most previous studies focus on static analysis, this study mainly provides a dynamic analysis of efficiency. The output of government health care expenditure has many different aspects, and so DEA is used to evaluate a group of DMUs with multiple inputs and outputs. We select the output orientation and Malmquist productivity concept that originated in the Malmquist index [40]. The earliest Malmquist index is obtained by DEA, and the Malmquist index is decomposed into two aspects: the first is evaluation DMU during two periods in the change of technical efficiency, and the second is the change of production technology, which reflects changes in the production frontier in DEA analysis [40]. The reference of adjacent joint frontiers is adopted, and the "DMU" of two adjacent periods is used to jointly construct a common front. The Malmquist index can be decomposed into efficiency (EC) and technology change (TC):

$$M(x^{t+1},y^{t+1},x^{t},y^{t})=\frac{E^{t\cup (t+1)}(x^{t+1},y^{t+1})}{E^{t\cup (t+1)}(x^{t},y^{t})}=\frac{E^{t+1}(x^{t+1},y^{t+1})}{E^{t}(x^{t},y^{t})}(\frac{E^{t\cup (t+1)}(x^{t+1},y^{t+1})}{E^{t+1}(x^{t+1},y^{t+1})}\frac{E^{t}(x^{t},y^{t})}{E^{t\cup (t+1)}(x^{t},y^{t})})=EC\times TC$$ (1)

*x*^*t*+1^,*y*^*t*+1^ mean the input indicator and output indicator in the period; t+1, *x^t^,y^t^* mean the input indicator and output indicator in the period t; *E*^*t*∪(*t*+1)^(*x*^*t*+1^,*y*^*t*+1^) means the MI in the period; t+1;*E*^*t*∪(*t*+1)^(*x^t^,y^t^*) mean the MI in the period t; $\frac{E^{t+1}(x^{t+1},y^{t+1})}{E^{t}(x^{t},y^{t})}$ means the EC and; $\frac{E^{t\cup (t+1)}(x^{t+1},y^{t+1})}{E^{t+1}(x^{t+1},y^{t+1})}\frac{E^{t}(x^{t},y^{t})}{E^{t\cup (t+1)}(x^{t},y^{t})}$ means the TC. The meaning of the Malmquist Index follows: greater than 1 indicates a productivity increase, while less than 1 is a productivity decrease; a technological change greater than 1 indicates technological progress, and a technological change less than 1 indicates technological decrease.

Färe et al. use an enhanced decomposition of the Malmquist index [40], which divides the efficiency change into the constant returns to scale technology change, pure technology efficiency change and a residual scale that captures changes in the deviation between variable returns and constant returns to scale technology [43]. The FGNZ [43] decomposition method is based on FGLR [40] decomposition, and further decomposes EC into PEC (Pure efficiency change or pure technical efficiency change) and SEC (Scale efficiency change).

This study calculates the TFP of provincial government’s health care expenditure by drawing on the panel data of 31 provinces in China in the period 2004–2019. The model calculates a set of Malmquist TFP and its decomposition indexes: technical efficiency change (TEC) and scale efficiency change (SEC);

MI=EC*TC=PEC*SEC*TC (2)

### 2.2 Variable selection

We take 31 provinces in China as 31 DMUs. The input variable is the provincial government health care expenditure in the period 2004–2019; output variables include the three health resources that represent the supply capacity of health care services, the number of health institutions, the number of beds in health institutions and the number of health care technicians (See Table 1).

**Table 1:** Definition of the variables.

Malmquist DEA model Variables		Definition
Input	Provincial government health expenditure	Government health expenditure refers to the funds that governments, of all levels, use for various purposes, including medical and health services, medical security subsidies, health and medical security management and population and family planning affairs.
Output	Medical and health institutions	The units that obtain the license of medical institution practice from the health administration department or obtain the registration certificate of legal person unit from the civil affairs, industrial and commercial administration department. They obtain the permission of the organization establishment administration department to provide medical care, disease control, health supervision services or engage in medical scientific research and medical on-the-job training. Medical and health institutions include hospitals, primary medical and health institutions, specialized public health institutions and other medical and health institutions.
	Health technicians	Health workers in primary hospitals, medical and health institutions, professional public health agencies and other institutions focused on medical and health work. ® Workers include health technicians, rural doctors and medical corpsman and other technical personnel, management personnel and logistical personnel. All are paid an end-of-the-year salary and are included in employee statistics.
	Beds of the medical institution	The number of beds refers to fixed existing beds (non-established beds) at the end of the year, including regular beds, cots, monitoring beds, beds in the process of disinfection and repair and beds discontinued due to expansion or overhaul. This category excludes neonatal beds for obstetrics, waiting beds for delivery in delivery room, stock beds, observation beds, temporary extra beds and patient home care beds.
Tobit regression model Variables		Definition
Region		In this study, regions are divided into the eastern (Beijing, Tianjin, Hebei, Liaoning, Shanghai, Jiangsu, Zhejiang, Fujian, Guangdong, Hainan, Shandong), central (Shanxi, Jilin, Heilongjiang, Anhui, Jiangxi, Henan, Hubei, Hunan) and western (Sichuan, Chongqing, Guizhou, Yunnan, Tibet, Shaanxi, Gansu, Qinghai, Ningxia, Sinkiang, Guangxi, Inner Mongolia) regions, and these distinctions are made on the basis of China’s seventh Five-Year Plan.
Economic		Per Capita GDP
Education		Illiterate Population Aged 15 and Over (by Region)
Urbanization		Proportion of Urban Population at Year-end (by region)
Population		Population density by (region)
Related policy		Health care interventions since 2009

### 2.3 Panel data Tobit model

The Malmquist DEA model focuses on the efficiency of the health care input and output, but does not consider some other relevant influential factors. The DEA-Tobit two-stage analysis framework is generally used to deal with this problem [44]. In the first stage, the DEA model is used to calculate the efficiency score of each DMU. The second stage is the regression of efficiency score that considers various relevant factors. Moran and Jacobs apply a two-stage DEA model that analyzes the efficiency of healthcare systems in 32 OECD countries, and then determine the relationship between the environmental factors and the efficiency performing Tobit regression analysis [45]. Medin evaluates the efficiency of hospitals in Nordic countries by applying the DEA and Ordinary Least Square (OLS) regression analysis methods [46].

We draw on these studies [1,44,47,48] and select related factors in population, economy and society. We select per capita GDP, the illiterate population aged 15 and over (by region), the proportion of the urban population at the end of the year (by region), population density (by region), application of the NHCR and per capita provincial government health care expenditure since 2009. We add dummy variables that represent the three regions into the regression equation to test if there are regional differences in the efficiency of government health care expenditure The regions are then divided into the eastern (Beijing, Fujian, Guangdong, Hainan, Hebei, Jiangsu, Liaoning, Shanghai, Shandong, Tianjin, Zhejiang,), central (Anhui, Heilongjiang, Henan, Hubei, Hunan, Jiangxi, Jilin, Shanxi,), and western region (Chongqing, Guizhou, Gansu, Guangxi, Inner Mongolia, Ningxia, Qinghai, Yunnan, Shaanxi, Sichuan, Sinkiang, Tibet), in accordance with China’s seventh Five-Year Plan.

The data type is balanced panel data and the dependent variable is the comprehensive technical efficiency score between 0 and 1. In order to make full use of the cross-section and time series information contained in the panel data and avoid errors caused by OLS estimation, we use the limited Tobit random effects panel model to conduct regression analysis. In order to establish the efficiency score of government health care expenditure for 31 Chinese provinces in the period 2004–2019, we incorporate panel data into the Panel Data Tobit Model:

$$Y_{it}={x\prime }_{it}\beta +\alpha_{i}+\varepsilon_{it}$$ (3)

*Y_it_* is the TC of the 31 provinces in China in the period 2004–2019; *α_i_* is the individual heterogeneity that is not observed and does not change over time; *x*′_*it*_ is the TC’s impact on social, economic and policy variables, including population density (people/km2), per capita GDP (yuan(¥)/person), illiterate population aged over 15 years-of-age, illiteracy rates, the fiscal decentralization index, health policy virtual variables, the features of the three regions and regional interaction between virtual and policy variables. The data type is balanced panel data and the dependent variable is the comprehensive technical efficiency score between 0 and 1. In order to make full use of the cross-section and time series information contained in the panel data and avoid errors caused by OLS estimation, we use the limited Tobit Random Effects Panel Model to conduct regression analysis.

### 2.4 Data source and statistical analysis

We use panel data from 31 provinces for the period 2004–2019. Data are divided before (2004–2009) and after (2010–2019) the NHCR. SPSS 22.0 is used to describe input and output in 31 of China’s provinces. The Malmquist DEA measurement process is calculated by MaxDEA8.18, and the Tobit Regression Model is calculated by STATA 16.0.

## 3. Result

### 3.1 Basic information on input and output

With regard to the degree of regional economic development, the eastern region is the richest, the central region is moderate and the western region is the worst-off. In the period 2003–2019, the health care expenditure of the provinces increased every year, and the growth rate of the eastern region was larger than that of its central and western counterparts. On the whole, the total medical input and output (including provincial health care expenditure, number of health care beds and the number of health care technicians) are higher in the eastern region than in the central and western regions.

### 3.2 First stage analysis: Total factor productivity

By drawing on the output-oriented Malmquist DEA model, we calculate the TFP for health care expenditure by the 31 provincial governments. Table 2 showed the TFP of the 31 provinces before health care reform was implemented in the period 2005–2009. With or without the implementation of NHCR, the average TFP of 31 provinces was lower than 1, which indicated the efficiency of China’s provincial health care expenditure was inefficient. The average TFP of 31 provinces increased after NHCR implementation, which indicates it improved the efficiency of provincial health care expenditure (See Tables 2 and 3).

**Table 2:** Total factor productivity effectivity of the 31 provinces in China before the new healthcare reform (2005–2009).

Region	MI	EC	TC	PEC	SEC
Eastern region	0.8559	1.0754	0.7959	1.0097	1.0650
Central region	0.7993	0.9871	0.8098	0.9816	1.0057
Western region	0.8343	1.0254	0.8136	1.0277	0.9978
Tibet	0.9748	1.1643	0.8372	1.2275	0.9486
Shanxi	0.8982	1.0875	0.8259	1.0774	1.0094
Fujian	0.8962	1.0907	0.8217	1.0877	1.0028
Guangdong	0.8937	1.1284	0.7920	0.9981	1.1306
Hebei	0.8913	1.0883	0.8190	1.0311	1.0555
Zhejiang	0.8902	1.1122	0.8004	0.9838	1.1305
Shandong	0.8868	1.0792	0.8218	1.0000	1.0792
Guizhou	0.8778	1.0668	0.8228	1.0718	0.9954
Jiangsu	0.8772	1.1118	0.7890	0.9717	1.1442
Qinghai	0.8723	1.0592	0.8235	1.0619	0.9975
Shanghai	0.8669	1.1100	0.7810	1.0350	1.0725
Guangxi	0.8633	1.0609	0.8137	1.0510	1.0094
Yunnan	0.8610	1.0593	0.8127	1.0082	1.0507
Beijing	0.8516	1.1223	0.7588	0.9986	1.1238
Tianjin	0.8355	1.1011	0.7588	1.1392	0.9665
Henan	0.8345	1.0165	0.8209	1.0000	1.0165
Ningxia	0.8276	1.0025	0.8255	1.0000	1.0025
Inner Mongolia	0.8080	1.0147	0.7963	1.0169	0.9979
Sinkiang	0.8034	1.0279	0.7816	1.0419	0.9865
Hunan	0.8020	0.9774	0.8205	0.9834	0.9938
Hubei	0.7974	0.9947	0.8016	0.9789	1.0162
Sichuan	0.7947	0.9871	0.8051	0.9899	0.9972
Chongqing	0.7926	0.9818	0.8073	0.9827	0.9991
Shaanxi	0.7850	0.9686	0.8104	0.9703	0.9983
Heilongjiang	0.7830	0.9710	0.8064	0.9636	1.0077
Jiangxi	0.7776	0.9673	0.8039	0.9664	1.0010
Gansu	0.7713	0.9311	0.8283	0.9371	0.9936
Hainan	0.7703	0.9730	0.7917	0.9620	1.0115
Liaoning	0.7688	0.9328	0.8242	0.9179	1.0162
Anhui	0.7671	0.9502	0.8073	0.9440	1.0066
Jilin	0.7446	0.9399	0.7922	0.9452	0.9944
Minimum	0.7446	0.9311	0.7588	0.9179	0.9486
Maximum	0.9748	1.1643	0.8372	1.2275	1.1442
Mean	0.8326	1.0327	0.8063	1.0092	1.0232
Degree of efficiency>1	0	19	0	15	19

**Table 3:** TFP of the 31 provinces in China after the NHCR (2010–2019).

Region	MI	EC	TC	PEC	SEC
Eastern region	0.9094	1.0098	0.9006	1.0094	1.0004
Central region	0.9139	1.0078	0.9068	1.0129	0.9950
Western region	0.9099	1.0052	0.9053	1.0058	0.9993
Heilongjiang	0.9637	1.0510	0.9169	1.0515	0.9996
Liaoning	0.9574	1.0457	0.9155	1.0447	1.0010
Yunnan	0.9470	1.0249	0.9240	1.0280	0.9970
Jilin	0.9428	1.0401	0.9065	1.0411	0.9990
Beijing	0.9332	1.0390	0.8981	1.0384	1.0005
Chongqing	0.9310	1.0064	0.9250	0.9976	1.0089
Zhejiang	0.9270	1.0297	0.9003	1.0294	1.0003
Sinkiang	0.9269	1.0043	0.9229	1.0003	1.0039
Anhui	0.9186	1.0047	0.9144	1.0143	0.9905
Jiangsu	0.9177	1.0135	0.9055	1.0127	1.0008
Hubei	0.9164	1.0054	0.9115	1.0114	0.9940
Inner Mongolia	0.9159	1.0216	0.8966	1.0225	0.9992
Shaanxi	0.9153	1.0181	0.8990	1.0176	1.0005
Tianjin	0.9150	1.0171	0.8996	1.0151	1.0020
Ningxia	0.9119	1.0102	0.9027	1.0000	1.0102
Shanghai	0.9117	1.0116	0.9012	1.0092	1.0023
Hunan	0.9075	1.0014	0.9062	1.0084	0.9930
Sichuan	0.9067	1.0015	0.9054	1.0051	0.9964
Guizhou	0.9061	0.9966	0.9092	0.9996	0.9970
Gansu	0.9045	1.0076	0.8977	1.0099	0.9977
Shandong	0.8991	1.0000	0.8991	1.0000	1.0000
Guangxi	0.8949	0.9946	0.8997	0.9943	1.0003
Shanxi	0.8941	0.9976	0.8962	1.0000	0.9976
Henan	0.8934	0.9899	0.9026	1.0000	0.9899
Qinghai	0.8904	0.9895	0.8999	0.9960	0.9934
Fujian	0.8903	0.9868	0.9022	0.9849	1.0019
Hebei	0.8883	1.0000	0.8883	1.0000	1.0000
Hainan	0.8857	0.9880	0.8965	0.9675	1.0212
Guangdong	0.8805	0.9784	0.9000	1.0041	0.9744
Jiangxi	0.8773	0.9748	0.9000	0.9782	0.9965
Tibet	0.8711	0.9878	0.8819	1.0000	0.9878
Minimum	0.8711	0.9748	0.8819	0.9675	0.9744
Maximum	0.9637	1.0510	0.9250	1.0515	1.0212
Mean	0.9107	1.0075	0.9040	1.0089	0.9986
Degree of efficiency>1	0	21	0	23	16

### 3.3 First stage analysis: The decomposition of the TFP (EC, TC)

After NHCR implementation, the average value of TC in the eastern, central and western regions increased. Irrespective of if NHCR was implemented, the EC of each province was found to be greater than TC. Meanwhile, the average value of EC exceeded 1, and the average value of TC was found to be less than 1. Before the implementation of NHCR, the average value of EC in 19 provinces was greater than 1; however, the average value of EC in 21 provinces was greater than 1 after NHCR implementation. Irrespective of NHCR, the average value of TC in 31 provinces was less than 1. This indicates that, in each province, health financial expenditure committed to technology was not sufficient to maintain technological development at a high level. But the average value of TC increased after NHCR implementation, suggesting that while NHCR policies promoted technological improvement, this still fell short of the efficiency frontier.

### 3.4 First stage analysis: The decomposition of the efficiency change (PEC, SEC)

In this study, the EC value is decomposed into PEC and SEC, (EC = PEC*SEC). Before NHCR implementation, average PEC in the eastern and central regions was less than the average change of SEC, while average PEC in the western regions exceeded the average SEC. The average PEC in 15 provinces was greater than 1, and the average SEC in 19 provinces was greater than 1. After NHCR implementation, the average PEC in the eastern, central and western regions were all higher than the average SEC. The average PEC in 23 provinces was greater than 1, confirming an increase of 6 provinces after NHCR implementation. The average SEC of the 16 provinces was greater than 1, indicating that NHCR is more focused on improving technical efficiency than expanding scale.

### 3.5 Second stage analysis: The result of the Tobit regression

The results of Tobit regression analysis were assumed to make it possible to analyze factors that affect the efficiency of the health care system. Because the TFP obtained by dynamic Malmquist DEA was the dynamic change data, MI should be taken as the dependent variable and data of dynamic change should be the independent variable; however, this is not possible as the dependent variable’s dynamic data cannot be obtained. Overall efficiency scores obtained from the static DEA base by the BCC model are therefore taken as the dependent variable. Table 4 shows the estimated results of Tobit regression model. Per capita GDP, population density and NHCR implementation are found to have a statistically significant effect on the efficiency of the provincial government’s health care expenditures (P<0.05). But region, education level, urbanization rate and per capita provincial financial expenditure are not found to be statistically significant (See Table 4).

**Table 4:** The result of the Tobit regression.

Related variables	Coef.	Std. Error	p	95%[Conf. Intercal]	
Region	-0.0146171	0.0131126	0.265	-0.0403773	0.0111432
Per Capita GDP	9.90e-07	4.62e-07	0.032	8.32e-08	1.90e-06
Illiterate Population Aged 15 and Over by Region	-3.69e-07	5.68e-07	0.516	-1.49e-06	7.47e-07
Proportion of Urban Population at Year-end by region	-0.0000608	0.0000468	0.194	-0.0001527	0.0000311
Population density by region	-1.252855	0.1600585	0.000	-1.567296	-0.9384142
New health care conduction	0.0753261	0.0282806	0.000	0.0197679	0.1308843
Government health expenditure	3.34e-06	2.95e-06	0.258	-2.45e-06	9.13e-06
_cons	0.6746997	0.0495504	0.000	-.5773562	0.7720433

## 4. Discussion

Rapid economic growth in China has increased public interest in health results. Although government health care expenditure increases every year, there are still a number of problems that require an effective response. The efficiency of the provincial government’s health care expenditure and influencing factors should therefore be studied in closer detail. This study utilized the DEA to assess the efficiency of government health care expenditure in 31 provinces, and performed the Tobit regression to explore the factors that influences government health care expenditure. The results directly address the three research questions. They demonstrate: (1) the efficiency of Chinese government health care expenditure in provinces in the eastern, central and western regions improved after the lunch of NHCR; (2) the efficiency of the eastern, central and western regions are not obvious different, and the TFP of the central region is relatively high. EC and PEC are efficient in the three regions, while TC is inefficient. The SEC of the eastern region is valid, while the SEC of the western and central region are invalid, and this shows that the TC still needs to be improved after NHCR; furthermore, the central and western regions need to give careful consideration to scale efficiency. The eastern region is however a developed region with more health care investment, and it also has a large population and high-quality health resources, which means it has an efficient scale efficiency; (3) Otherwise, research shows that the efficiency of provincial government’s health expenditure is related to population density, per capita GDP and the implementation of NHCR.

Health care system efficiency has been extensively researched, but previous studies have a number of defects: firstly, they were mostly based on cross-sectional data for efficiency accounting, and this makes it impossible to analyze the trend of dynamic efficiency change in government health care expenditure [10,24,27,37,49,50]; secondly, a number of contributions to the literature take health outcomes, such as life expectancy and child mortality, as output variables when calculating the efficiency of health care system; however, various health care resources are the most direct output of government health care expenditure [51]. The complexity of the health decision process means that using health outcomes as an output of government health care expenditure will inevitably introduce more bias, and this will affect the accuracy of the accounting results. We therefore choose provincial health care expenditure as the input and select the number of health institutions, the number of beds in health institutions and the number of health care technicians as direct variables. They are then used to present the health care output. The main benefit of this approach is that it is consistent with the aforementioned studies [44,52].

This study shows that the efficiency of Chinese government health care expenditure has improved since the implementation of NHCR. Since this process began in 2009, the Chinese government has increased its health care investment, paid increasing attention to medical and health care, and has also implemented a series of reform measures with the aim of solving existing problems. This finding is consistent with the research results of Guan Yongbin, Zhao Yinyin, Zhang Zhongfang and Chen Shiyi, who claim that the launch of NHCR can effectively promote the improvement of government health care efficiency, and also that China should persist with relevant NHCR policies [1,44,53,54]. For instance, Guan Yongbin et al studied the efficiency of China’s medical and health care expenditure from 2009 to 2011 and Zhao Yinyin et al studied the performance of government health investment from 2010 to 2017. And their results consistently found that the efficiency of China’s medical and health financial expenditure significantly improved after the NHCR. However, these studies also show that the efficiency of Chinese government health care expenditure is still not fully efficient, and also indicate that the average level is yet efficient and effective. This also suggests that government should pay much more attention to TC and improve the efficiency of government health expenditure by optimizing management and strengthening fund management.

With regard to the efficiency of the eastern, central, and western regions, our findings are inconsistent with Guan Yongbin and Zhao Yinyin. In this study, we found there was no obvious difference between the eastern, central, and western region in relation to efficiency, although the central region scored relatively highly.

Guan Yongbin found that the efficiency of government health care expenditure showed significant differences across separate regions, and also noted the eastern region when compared with the central and western regions, had a higher relative efficiency level [55]. This was because Guan Yongbin used the standard DEA that analyses static efficiency; however, the efficiency changes when the Malmquist DEA is used. The inconsistency of the research results mainly lies in the different connotations and emphases presented by the two aspects. Otherwise, Tobit regression showed that efficiency is not related to the regions. This study uses the Malmquist DEA to evaluate and compare the efficiency of provincial government health care expenditure in the period 2004–2019. The TFP, EC, TC, PEC and SEC in the period 2004–2019 are analyzed. The results show that the average TFP of provincial government health care expenditure was always lower than 1 in period 2004 to 2019; meanwhile, the average TFP of each province was lower than 1. This indicates that the overall average efficiency of government health expenditure is always low after NHCR. In each region, TFP improved after the implementation of NHCR, but it still failed to reach frontier efficiency. The average TFP of Tibet, Shanxi, Fujian, Guangdong and Hebei ranked in the top five in the country before the implementation of NHCR. However, the average TFP of the five provinces ranked behind the whole country, and Tibet showed a particularly sharp descent, dropping from first to last after NHCR implementation. Tibet, which is part of the western region, has low population density, low per capita GDP and an underdeveloped economy. Its level of regional economic development continues to limit government investment, the construction of medical institutions and the influx of medics to the western region. This is consistent with Liu Zimin (2014), who suggests that inefficiencies in the western region are affected by economic, geographic and social factors that include population density, per capita GDP, illiteracy, urban population proportion and total raising ratio [56]. Although the TFP of these five provinces decreased, the TC increased after NHCR implementation. However, the PEC of these 5 provinces were lower than the SEC, and this indicates they did not pay attention to technological improvement after NHCR. The SEC in the other four provinces, with the exception of Tibet, increased after NHCR. Irrespective of NHCR, the TFP of Zhejiang and Jiangsu ranked first of the 31 provinces. Meantime, the TC and PTC of Zhejiang and Jiangsu improved after NHCR, and this may be related to their focus on technological improvement. Jiangsu and Zhejiang provinces are part of the eastern developed regions, in the term of the construction of medical institutions, the number of health and technical personnel, and health management, has a congenital advantage. When the government increases its investment, the two provinces will be able to catch up, reasonably increase their input and output, and focus on technological change and improve managerial efficiency. This will enable the two provinces to maintain a high level of efficiency.

This study found that per capita GDP, population density and the implementation of NHCR are related to government health care expenditures. These findings are consistent with the conclusions of Han Huawei [52], who claims that per capita GDP helped to promote government health care expenditure. With regard to demographic characteristics, Grossman claims that the costs of management and supervision are negatively correlated with an area’s population density, and claims this leads to the problem of economies of scale [57]. This means that the provision of public services to an increasing number of residents will produce economies of scale, which will in turn produce improvements in spending efficiency.

Athanassopoulos et al find that population density had a negative impact on government efficiency [58], but this is challenged by Chen Shiyi, who claims that it is positively correlated with government health care expenditure [44]. This confirms that when more people are in an area, the scale economy effect of government expenditure will be more significant. In addition, it will also be more convenient for local government to organize and provide a public consumption service network. But Han Huawei, in referring to government health care expenditure, claims that: 1) population density has a weak negative impact; 2) fiscal decentralization has a negative impact; and 3) a high illiteracy rate helps to promote government health care expenditure [52]. The influence of population factors on the efficiency of government public expenditure is mainly manifested in population density and the urban and rural population structure.

Theoretically speaking, when a region has a larger population per unit area, the local public service network will be closer, the scale economy effect of government public expenditure will be more significant and government public expenditure will be higher. But when a region has a higher urbanization level, the scale economy effect of government public expenditure will be more significant, and this may be more conducive to improvements in the efficiency of government expenditure [54]. Qin Shikun shows that per capita GDP, urbanization rate and population density do not significantly affect total factor productivity [59]. Some researcher find that education can improve the efficiency of local government health care expenditure in a positive way [60,61], and it is generally believed that higher levels of per capita GDP or wealth will positively affect spending efficiency because wealthier residents will put more pressure on local authorities to meet the level of demand for efficient public services [54]. But Eeckaut and DeBorger suggest that high-income areas will lead the government to support idle workers, meaning that the government will lose the incentive to introduce control costs and efficiency will worsen [62].

Migue and Belanger find that the efficiency of government expenditure can be improved if variables that help to explain residents’ ability to supervise the government are introduced [63]. Qin Shikun claims that fiscal decentralization and the improvement of the education level are positively correlated with the TFP of government medical and health care expenditure, and also asserts that the illiteracy rate is negatively correlated with the TFP of government medical and health care expenditure.

## Conclusion

This study provides a number of clear policy recommendations. First, with regard to fiscal policy, local governments in eastern China should seek to eliminate efficiency loss by optimizing government expenditure structure; provinces in western China, meanwhile, should increase government health investment with the aim of improving their efficiency.

Within the existing institutional framework, moderate fiscal centralization will help improve the efficiency of government health care expenditure, and the transfer of fiscal expenditure responsibility to the higher level of government should therefore be considered.

Second, from a health policy perspective, a deepened reform of the health care system and the rational allocation of various health resources will help to improve the efficiency of health care expenditure in China. Last, but by no means least, China should continue to implement the NHCR. However, it should not just focus on final expenditure but should give much greater consideration to technological reform.

## Supporting information

S1 Dataset (XLS)

S2 Dataset (XLS)

## Abbreviations

NHCR: New Health Care Reform
DEA: Data Envelopment Analysis
DMU: Decision Making Unit
TFP: Total Factor Productivity
EC: Efficiency Change
TC: Technology Change
PEC: Pure Efficiency Change/Pure Technical Efficiency Change
SEC: Scale Efficiency Change
TE: Technical Efficiency
GDP: Gross Domestic Product
CCR: Charnes Cooper Rhodes
BCC: Banker Charnes Cooper
CRS: Constant Returns to Scale
VRS: Variable Returns to Scale
OLS: Ordinary Least Square
